# Yeasts affect tolerance of *Drosophila melanogaster* to food substrate with high NaCl concentration

**DOI:** 10.1371/journal.pone.0224811

**Published:** 2019-11-06

**Authors:** A. S. Dmitrieva, S. B. Ivnitsky, I. A. Maksimova, P. L. Panchenko, A. V. Kachalkin, A. V. Markov

**Affiliations:** 1 Department of Biological Evolution, Faculty of Biology, Lomonosov Moscow State University, Moscow, Russia; 2 Department of Soil Biology, Faculty of Soil Science, Lomonosov Moscow State University, Moscow, Russia; 3 G.K. Skryabin Institute of Biochemistry and Physiology of Microorganisms, Russian Academy of Sciences, Pushchino, Russia; 4 Borissiak Paleontological Institute, Russian Academy of Sciences, Moscow, Russia; Inha University, REPUBLIC OF KOREA

## Abstract

The ability of model animal species, such as *Drosophila melanogaster*, to adapt quickly to various adverse conditions has been shown in many experimental evolution studies. It is usually assumed by default that such adaptation is due to changes in the gene pool of the studied population of macroorganisms. At the same time, it is known that microbiome can influence biological processes in macroorganisms. In order to assess the possible impact of microbiome on adaptation, we performed an evolutionary experiment in which some *D*. *melanogaster* lines were reared on a food substrate with high NaCl concentration while the others were reared on the standard (favourable) substrate. We evaluated the reproductive efficiency of experimental lines on the high salt substrate three years after the experiment started. Our tests confirmed that the lines reared on the salty substrate became more tolerant to high NaCl concentration. Moreover, we found that pre-inoculation of the high salt medium with homogenized salt-tolerant flies tended to improve reproductive efficiency of naïve flies on this medium (compared to pre-inoculation with homogenized control flies). The analysis of yeast microbiome in fly homogenates revealed significant differences in number and species richness of yeasts between salt-tolerant and control lines. We also found that some individual yeast lines extracted from the salt-tolerant flies improved reproductive efficiency of naïve flies on salty substrate (compared to baker’s yeast and no yeast controls), whereas the effect of the yeast lines extracted from the control flies tended to be smaller. The yeast *Starmerella bacillaris* extracted from the salt-tolerant flies showed the strongest positive effect. This yeast is abundant in all salt-tolerant lines, and very rare or absent in all control lines. The results are consistent with the hypothesis that some components of the yeast microbiome of *D*. *melanogaster* contribute to to flies’ tolerance to food substrate with high NaCl concentration.

## Introduction

Experimental evolution is a research approach which is increasingly used to analyze the functioning of fundamental evolutionary mechanisms (mutations, selection, drift) in real time in controlled conditions. Several experimental evolution studies have shown the ability of model organisms, such as the fruit fly *Drosophila melanogaster*, to adapt quickly to various adverse conditions [1]. It is usually assumed by default that the observed adaptation to a new environment is explained by changes in the gene pool of the experimental population, or by more transient epigenetic changes. Meanwhile, it is known that microbiome plays an important role in the lives of many animals; it can be transmitted from parents to offspring [2] and influence the fitness of macroorganisms in particular environmental conditions [3]. Thus, within the framework of the so-called “hologenomic theory of evolution,” which is rapidly gaining popularity among evolutionary biologists, it is proposed that the basic unit of selection is not a separate organism, but a holobiont [4], i.e. a system consisting of a macroorganism and its microbiome [3, 5–7]. *D*. *melanogaster* is a well-studied species with a relatively simple microbiome and well-developed genetic tools. It is considered a good model for investigating host–microbe interactions [8–10].

Until very recently, there was almost no direct experimental evidence that the observed increase of fitness of model animals in evolutionary experiments can be explained to some extent by changes in the microbiome, rather than by changes in the macroorganism itself. Nonetheless, many facts are compatible with this assumption. For instance, experiments on “artificial speciation” of aphids performed in the mid-20th century have shown that transfer to a different plant species can result in rapid emergence of partial reproductive isolation in these insects [11]. At that time, this unexpected result was not properly explained. Later, however, such obligate symbionts of aphids as *Buchnera* were discovered; these bacteria provide aphids with adequate nutrition [12, 13]. It is likely that adaptation of aphids to the new host plants, as well as some other adaptive abilities (e.g., temperature optimums), depend on the evolution of the endosymbionts [14]. Moreover, some experimental data implies that microbiome probably can influence mate choice, which, in turn, can facilitate rapid development of partial behavioral isolation [15–17], although recent researh casts some doubt on the validity of these conclusions [18, 19].

The ability of *D*. *melanogaster* to adapt quickly to adverse conditions, including food substrates with high NaCl concentrations, makes this species an appropriate object to study mechanisms of adaptation. Although *D*. *melanogaster* is not typically found on high salt substrates in nature, adaptation of this species to salty food proved to be a convenient experimental model [20–27]; moreover, there are numerous salt-adapted species in other Diptera families, e.g., Ephydridae [28].

Salt concentrations exceeding 2% are a negative factor for the wild type *D*. *melanogaster*, and concentrations higher than 4% can be fatal to larvae and adults [20, 21]. Nevertheless, several evolution experiments have demonstrated that laboratory lines of *D*. *melanogaster* are able to adapt to NaCl concentrations as high as 6–8% over several dozen generations, given that salt concentration increases gradually [20, 22–27]. Moreover, the evolved tolerance to salty food can result in higher reproductive efficiency of the flies not only on the salty substrate, but also on the standard, favourable food substrate [26]. These results do not contradict the assumption that flies' adaptation to salt is due to changes in microbiome. Such changes may probably act as a broad-scale adaptation, simultaneously increasing the fitness of the flies on various food substrates, although this possibility has not yet been experimentally tested.

Phenotypic plasticity under exposure of naïve *D*. *melanogaster* to salty food has been described, including anal papillae size reduction in larvae [20] and changes in expression levels of several genes involved in secretion [21]. Otherwise, little is known about the mechanisms of adaptation of *D*. *melanogaster* to high NaCl concentrations in the course of evolution experiments. To our knowledge, there are no data on the possible contribution of microbiome (bacteria and yeasts, carried by the flies on the cuticle and in the gut) to such adaptation. On the other hand, data on *D*. *melanogaster* microbiome (which has become a popular object of research in recent years) indicate that such a contribution is conceivable. It has been shown that microbiome modulates host metabolic gene expression, metabolic response to diet, the efficiency of food resource exploitation, immune response, adult life expectancy, and larval growth rate [29–36], and that host genetic control of microbiome affects the nutritional status and other physiological characteristics of the flies [36, 37]. Microbes can rescue undernutrition in *Drosophila* [38]. Moreover, microbiome species richness in *Drosophila* species correlates with the diet of the flies [39], while different species of yeasts present in the food substrate influence differently the survival of larvae and duration of larval development [40–42]. Flies carry bacteria and yeasts in their guts and on the body surface, and progeny feeding on a substrate on which their parents had lived can ensure transgenerational transmission (‘inheritance’) of the microbiome [32, 33].

In the current paper, we focused on the possible impact of yeasts on the adaptation of *D*. *melanogaster* to high salt diet, because we noticed that fly lines reared on salty food usually carry more yeasts than the lines reared on standard food. Further research is needed to assess the role of symbiotic bacteria as well.

The current study builds on two previous findings.

First, we have shown previously [26] that the two *Drosophila* lines (Fs1, Fs2) which have been reared on the salty substrate for 11 months became more tolerant to salty food and reproduced on this substrate more efficiently than the two control lines (Fn1, Fn2), which have been reared on the standard (favourable) substrate. The same four lines are used in the current study. Here we show that the lines Fs1, Fs2 are still more salt-tolerant than the lines Fn1, Fn2 after three years of the evolution experiment.

Second, in order to assess the possible impact of the microbiome on adaptation of *Drosophila* to high NaCl concentration, we have previously compared the reproduction efficiency of the flies on the high-salt food medium pre-inoculated with homogenized flies from either salt-tolerant or control lines. We found that pre-inoculation with homogenized flies from salt-tolerant lines improved reproduction of the flies on a high-salt medium compared to pre-inoculation by homogenized flies from the control lines. We obtained this result in four laboratory lines (two salt-tolerant and two control ones) reared in population cages with overlapping generations. We also found remarkable differences in the abundance and taxonomic composition of yeasts between salt-tolerant and control *Drosophila* homogenates [43, 44]. These results agree with the assumption that the increased tolerance of *D*. *melanogaster* to salty food as observed in evolutionary experiments is probably due to quantitative or qualitative changes in the microbiome which enhance the fitness of the flies (and the entire assemblage—holobiont) on salty food. Here we test the robustness and replicability of this result using four other *D*. *melanogaster* lines (Fs1, Fs2, Fn1, Fn2), maintained under different conditions (in glass vials with non-overlapping generations).

We also attempt, for the first time, to reveal specific components of microbiome that influence the fitness of *D*. *melanogaster* exposed to salty food. We show that yeast strains isolated from salt-tolerant fly lines (Fs1, Fs2) appear to enhance reproduction of naïve *D*. *melanogaster* on salty food more efficiently than yeast strains isolated from control lines (Fn1, Fn2).

**Fig 1:**
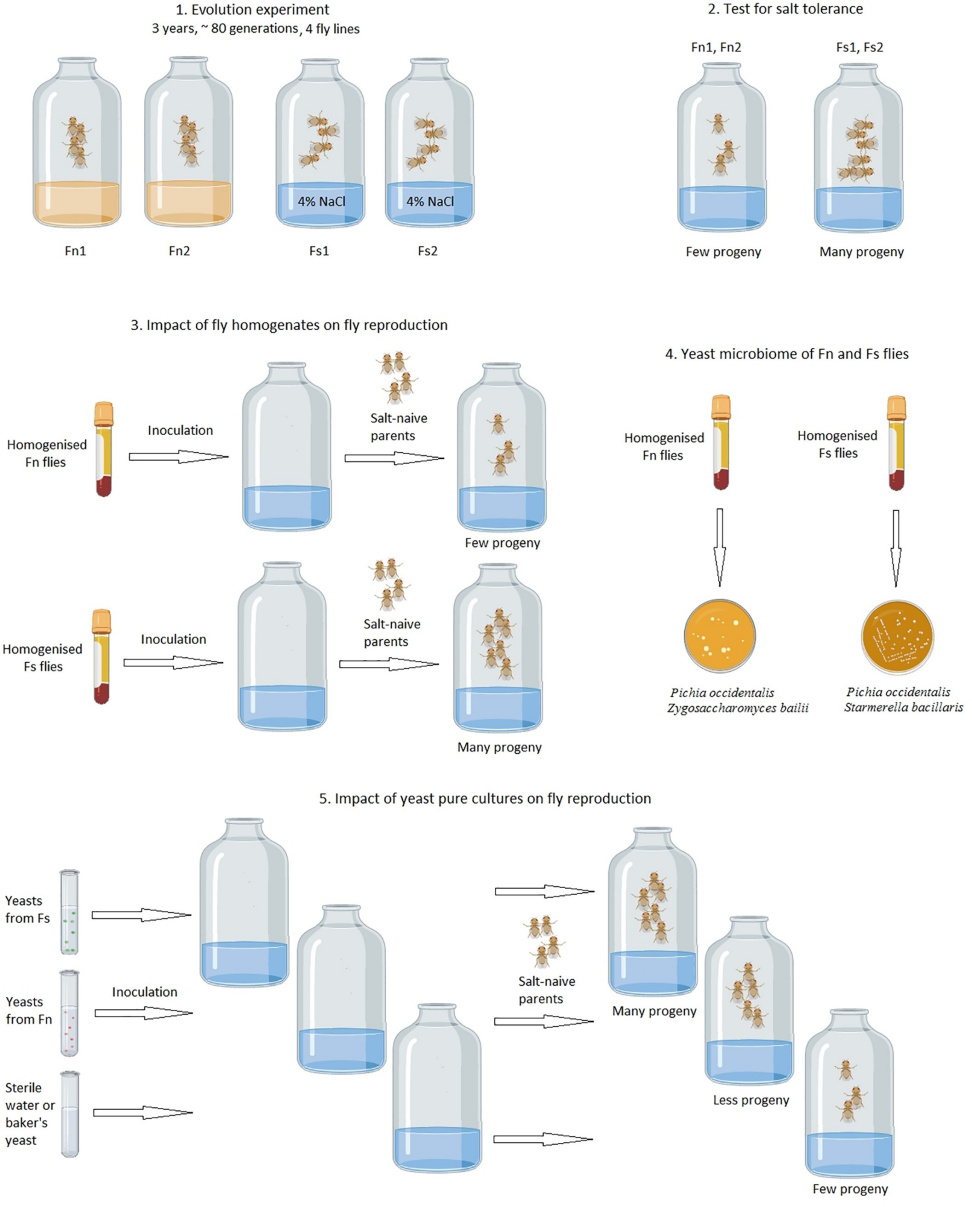
Schematics of the experimental design and the main findings. Fn1, Fn2: fly lines reared for three years on the standard (favourable) food medium; Fs1, Fs2: lines reared for three years on the high salt medium.

The study included four steps (Fig 1):

1. First, we ascertained that the two *Drosophila* lines (Fs1, Fs2) which have been reared on the salty substrate for three years in the course of our evolution experiment, are more tolerant to salty food and reproduce on this substrate more efficiently than the two control lines (Fn1, Fn2), which have been reared on the standard (favourable) substrate. We had tested the same four lines previously, 11 months after the start of the evolution experiment, and obtained positive result [26]. In the current study, we show that this result is replicable after three years from the start of the experiment.
2. Next, we confirmed that pre-inoculation of the high salt medium with homogenized salt-tolerant flies (Fs1, Fs2) improved the reproduction of control (naïve) flies on this medium compared to pre-inoculation by homogenized flies from the control lines (Fn1, Fn2). Thus we show that the results obtained previously [43, 44] are replicable in four other fly lines, reared in different conditions (see above).
3. We evaluated the abundance and taxonomic composition of yeasts in the four experimental lines of *D*. *melanogaster*. Pure cultures were obtained from five yeast strains found in either control or salt-tolerant lines (three strains from Fs1 and two strains from Fn1). We show that there are robust differences between yeast communities from salt-tolerant and control fly lines. Most importantly, one yeast species, *Starmerella bacillaris*, is abundant in all salt-tolerant lines tested to date (Fs1, Fs2, and two other salt-tolerant lines tested in [44]) and absent or very scarce in all control lines (Fn1, Fn2, and two other control lines tested in [44]).
4. Finally, we examined for the first time the influence of each of the five yeast strains on the fitness of naïve flies exposed to salty food. We found that strains isolated from the salt-tolerant flies improved the reproduction of the flies on salty food better than strains extracted from the control flies, and better than *Saccharomyces cerevisae*, the common bakers’ yeast (which is absent in the homogenates of the experimental flies). *Starmerella bacillaris* yeast demonstrated the strongest positive effect.

Thus, the results are compatible with the hypothesis that yeasts contribute to the adaptation of *D*. *melanogaster* to high salt substrates; we also identified components of the yeast microbiome which are likely to be responsible for this contribution.

## Material and methods

### Design of the evolutionary experiment and experimental populations

In the current study we used *D*. *melanogaster* lines which have been living for three years (about 80 generations) on one of the two food substrates: standard (favourable) or high salt (stressful).

The initial population of *D*. *melanogaster* was derived from 30 wild flies caught in southwestern Moscow (Russia) in September 2014. The evolutionary experiment started in October 2014 with *D*. *melanogaster* lines derived from the initial population and cultivated on different food substrates. Each line was derived from 30 flies randomly chosen from the initial population; no isofemale lines were established. The flies were reared at 23–24°C and natural lighting.

We used four lines in the current study:

Fn1, Fn2: lines reared on the standard laboratory food medium (60 g inactivated yeast, 35 g semolina, 50 g sugar, 45 g crushed raisins, 8 g agar, 2 g propionic acid per 1 L of food). Hereafter, this food medium is denoted by the letter N.

Fs1, Fs2: lines reared on a salty food medium (the medium N with addition of 40 g of NaCl per 1 L of food). Hereafter, this food medium is denoted by the letter S.

Populations were maintained in cylindrical glass vials, 64 mm in diameter and 100 mm in height, cotton-plugged, with 66.7 mL of food per vial. Each vial also contained a drinking bowl: a 1 mL cylindrical plastic reservoir filled with moist cotton wool. Each population occupied one vial. Every two weeks, all adults in the vial were immobilized by carbon dioxide; subsequently, 10 males and 10 females were selected at random and placed in a new vial with fresh food; the remaining flies were discarded.

### Tests on the efficiency of reproduction on different food substrates

In order to test the flies’ reproduction efficiency on different food substrates (with or without previous inoculation by yeasts or homogenized flies, see below), we placed 10 pairs of parents in a vial with food and kept them there for 7 days, after which the parents were discarded. Subsequently, we counted their offspring (adults and pupae) daily until the adult offspring ceased to emerge. We used the total number of adult offspring as a measure of reproduction efficiency.

We used the same testing technique previously in the course of our experiment [25–27, 43, 44]. We performed three tests on the reproductive efficiency (sections 1, 2 and 4 of the Results); 5 to 10 test vials were used for each fly line/substrate combination.

**In the first experiment** (section 1 of the Results), we evaluated the fitness of the flies from the four lines on the substrates N and S. There were eight fly line/substrate combinations (parents from the line Fn1 tested on food N (Fn1/N), Fn2/N, Fs1/N, Fs2/N, Fn1/S, Fn2/S, Fs1/S, Fs2/S). Parents were selected at random from the experimental populations.

**In the second experiment** (section 2 of the Results), we compared the reproduction efficiency of salt-naïve flies from the control line Fn1 on salty substrate pre-inoculated with homogenized flies from one of the four experimental lines. Inoculation was performed two days prior to placing the parents in the vial, so that microorganisms in the homogenate had time to reproduce. We used virgin males and females from the line Fn1 as parents.

**In the third experiment** (section 4 of the Results), we evaluated the reproduction efficiency of Fn1 flies on the salty substrate which have been previously (two days prior to placing the parents) inoculated with one of the yeast strains. We used five yeast strains extracted from the flies and two controls: bakers’ yeast *S*. *cerevisiae* and no yeast (sterile water); seven to eight vials were used for each variant. Virgin males and females from the line Fn1 were used as parents.

Virgin flies were immobilized by carbon dioxide within eight hours after eclosion, separated by sex and transferred to test tubes with food N. They were used in tests 8–12 days after eclosion. Tests were performed at 23–24°C and natural lighting in standard glass vials with 66.7 mL of food per vial.

To prepare the homogenate, 110 randomly selected flies from one of the four lines (Fn1, Fn2, Fs1, or Fs2), aged 0 to 5 days after eclosion, were immobilized by placing in a freezer at −20°C for 3 minutes. Thereafter, the flies were transferred to sterile test tubes with 1100 μl of sterile water (10 μl per fly, which results in approximately 1:10 dilution, because the average mass of an adult *D*. *melanogaster* is about 1 mg), mashed with a sterile glass rod and mixed for 15 minutes using a Heidolph Multi Reax vortex, at 1600 rpm. Subsequently, we took 100 μl of the homogenate for the analysis of yeast abundance and species composition (see below). The rest of the homogenate (1000 μl) was put onto the food substrate with a pipette with a sterile tip, 150 μl of the homogenate per vial, and evenly distributed with a small sterile glass spatula. Thus the amount of the homogenate applied to the food substrate in each vial corresponds to about 15 homogenized flies. Two days after the homogenate was applied to each vial, we placed 10 pairs of parents (10 females and 10 males) and removed them 7 days later; after that, we monitored the development of the offspring.

### Analysis of yeast composition in *D*. *melanogaster* homogenates

We studied yeast population of *D*. *melanogaster* lines Fn1, Fn2, Fs1 and Fs2 using the inoculation method on a dense growth medium. We added 900 μl of sterile water to 100 μl of the homogenate (described above) and mixed it using the vortex for three minutes. As a result, we had a dilution of 1 : 100 (one homogenised fly, weighing about 1 mg, per 1 mL of water). Using a pipette with a sterile tip, we placed the aliquot of the suspension (50 μl) on the surface of the dense growth medium GPYA (20 g/L glucose, 10 g/L peptone, 5 g/L yeast extract, 200 g/L agar, and 1 g/L levomycetin to prevent bacterial growth), spread in a sterile Petri dish. Subsequently, we evenly distributed the aliquot on the surface of the medium using a Drigalski spatula. We inoculated the fly suspension from each line (Fn1, Fn2, Fs1, Fs2) in tenfold replication (10 Petri dishes per line). Inoculated dishes were incubated for five days at a room temperature (20–22° C).

By the end of this period, all yeast colonies were divided into types by morphology and counted. For each sample, we obtained the overall number of yeasts in colony-forming units (CFU) per fly. We extracted two to three strains of each colonial morphotype to obtain pure cultures, then grouped all the cultures by cultural and micromorphology traits. The final identification of the groups was based on analysis of the nucleotide sequences of the ITS1-5.8S-ITS2 region and D1/D2 of rDNA domain 26S (LSU) using the method described previously [45].

Amplification of rDNA regions was performed using ITS1f (5’-CTTGGTCATTTAGAGGAAGTA) and NL4 (5’-GGTCCGTGTTTCAAGACGG) primers. The same primers were used for sequencing. We used an Applied Biosystems 3130xl Genetic Analyzer sequence by Syntol Scientific Production company (Moscow, Russia). Data analysis was performed using the NCBI (ncbi.nlm.nih.gov) or CBS database (www.cbs.knaw.nl). The obtained sequences were deposited in GenBank database (MK332454-MK332474).

### Use of yeast pure cultures for inoculation of the high salt medium

We prepared suspensions of the five most abundant yeast strains (extracted from *D*. *melanogaster* lines Fn1 and Fs1) by taking 7 mm^3^ pure culture biomass and 1 mL sterile water and mixing them for three minutes in the Multi Reax vortex at 1980 rpm. The suspensions were spread on the surface of the high salt medium, 150 μl per vial, with a pipette with sterile tips and small Drigalski spatulas, under sterile conditions. The vials were then incubated for two days at a room temperature (20–22° C). Subsequently, we placed 10 pairs of parents (10 females and 10 males) into each vial, removed them 7 days later, and monitored the development of the offspring.

## Results

### 1. Reproduction efficiency of *D*. *melanogaster* on the food substrates N and S

The aim of the first experiment was to estimate the fitness of the four *D*. *melanogaster* lines (two salt-tolerant and two control ones) on the substrates N and S. We had performed the same experiment previously, in September 2015, 11 months after the start of the evolutionary experiment. We had previously found that the flies from the lines Fs1 and Fs2 produced more progeny on both foods (N and S), compared to lines Fn1 and Fn2 [26].

**Fig 2:**
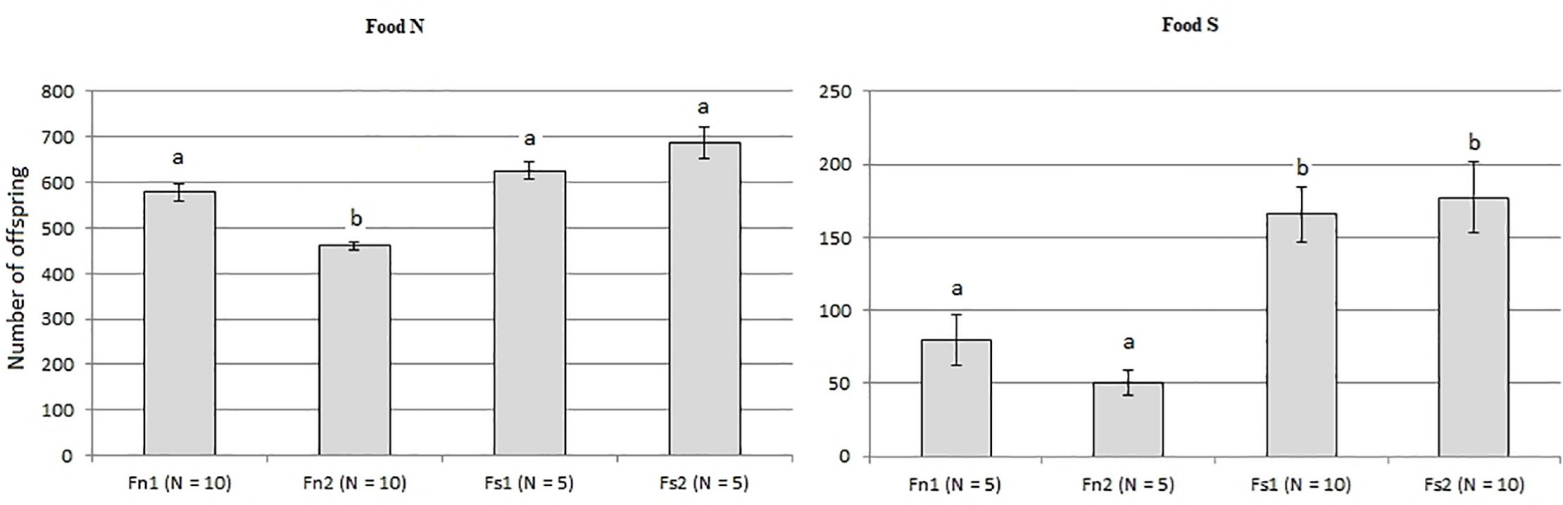
Reproduction efficiency of four *D*. *melanogaster* lines on foods N and S. Lines: Fn1, Fn2, control (reared on food N); Fs1, Fs2, salt-tolerant (reared on food S). The vertical axis shows the mean number of adult offspring produced by 10 pairs of parents (± SEM); N, number of vials in each test. Different letters indicate significant differences (p < 0.05, Dunn’s test).

Re-testing was performed in August 2017, 34 months after the start of the evolutionary experiment. The results are shown in Fig 2, raw data are available in S1 Table.

The figure shows that flies from the salt-tolerant lines (Fs1, Fs2) produced more offspring on salty food than flies from the control lines (Fn1, Fn2), which agrees with the earlier results [26]. On standard food N the results are less conclusive: Fn2 produced significantly less offspring than both Fs1 and Fs2, but the difference between Fn1 and the two salt-tolerant lines was insignificant.

The null hypothesis that all four lines belong to the same distribution is rejected (*p* = 0.000076, Kruskal–Wallis test) for the food N (Fig 2, left graph). Paired comparisons revealed significant differences between the line Fn2 and the other lines (for pairs Fn2/Fn1, Fn2/Fs1, Fn2/Fs2 *p-*value is 0.00500, 0.00062 and 0.00005, respectively; Dunn’s test). Other differences are insignificant.

For the salty food S (Fig 2, right graph), the null hypothesis that there are no differences between the lines is also rejected (p = 0.000862, Kruskal–Wallis test). All paired differences between control and salt-tolerant lines are statistically significant (for pairs Fn1/Fs1, Fn1/Fs2, Fn2/Fs1, Fn2/Fs2 *p-*value is 0.01281, 0.01909, 0.00091, 0.00151, respectively; Dunn’s test). Thus, flies that were reared on salty food demonstrate a significantly higher reproductive success on the salty substrate compared to the control flies.

The results are consistent with our previous conclusions that flies from the lines Fs1 and Fs2 have a higher fitness when exposed to both types of food; thus, their acquired tolerance to salt probably resulted in trophic niche expansion rather than specialization [26], although further experiments are required to test this possibility.

### 2. Influence of fly homogenates on the reproductive efficiency of *D*. *melanogaster* on salty substrate

The aim of the second experiment was to evaluate the influence of the microbiome of different fly lines on the reproductive efficiency of flies on salty food.

**Fig 3:**
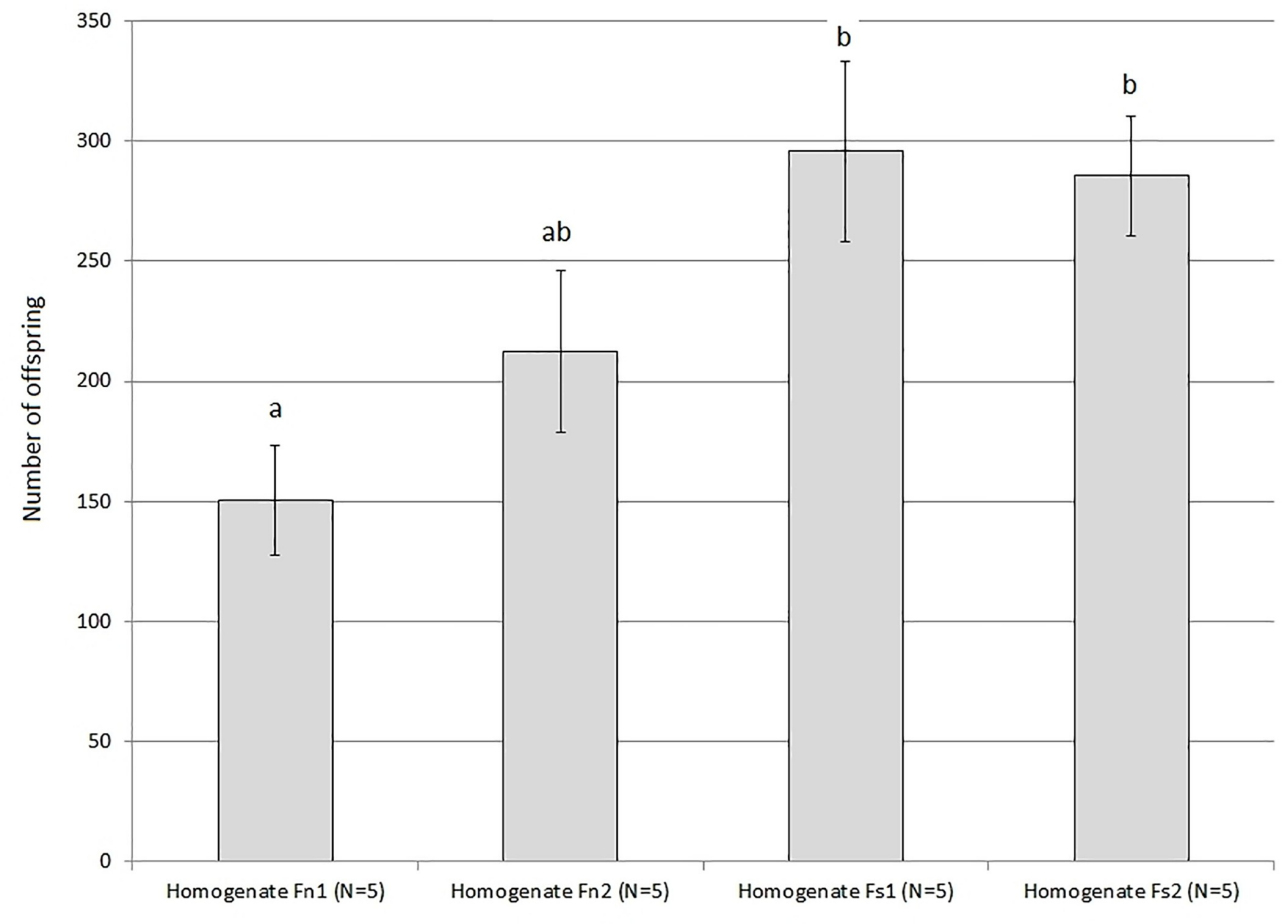
The influence of the control (Fn1, Fn2) and salt-tolerant (Fs1, Fs2) fly homogenates on the reproductive efficiency of Fn1 flies on food S. The vertical axis shows the mean number of adult offspring produced by 10 pairs of parents (± SEM); N, number of vials in each test. Different letters indicate significant differences (p < 0.05, Dunn’s test).

We compared the influence of pre-inoculation of food S with homogenized flies from the lines Fn1, Fn2, Fs1, Fs2 on the reproduction efficiency of the naïve (not salt-tolerant) flies from the control line Fn1. The experiment was performed in September–October 2017. The results are shown in Fig 3, raw data are available in S2 Table.

The figure shows that flies produced more offspring in the vials where the homogenate of the salt-tolerant flies (Fs1, Fs2) was spread over the surface of the salty substrate, compared to the vials inoculated by the homogenized control flies (Fn1, Fn2). This agrees with the results obtained previously in the four other *D*. *melanogaster* lines [43, 44].

The Kruskal–Wallis test rejects the null hypothesis that all four lines belong to the same distribution (*p* = 0.01979). The paired comparisons showed significant differences between Fn1 homogenate and both “salt-tolerant” homogenates (Fs1, Fs2); *p*-value is 0.00748 and 0.01024, respectively (Dunn’s test). In the rest of the cases, the differences are not significant (for pairs Fn2/Fs1 and Fn2/Fs2 *p*-value is 0.10854 и 0.13419, respectively), which may be partly due to small sample size (only five vials per test).

Thus we can conclude that the results previously obtained in four other *D*. *melanogaster* lines kept under different conditions (in population cages) have been successfully reproduced. We confirmed that the homogenate of salt-tolerant flies spread over the surface of the salty substrate improved the reproduction of *D*. *melanogaster* on such substrate compared to the homogenate of the control flies.

These results, together with the ones obtained earlier, imply that some characteristics of the microbiome of salt-tolerant flies may contribute to the observed increase in salt tolerance. It is also possible that additional factors independent of the different microbiome between Fn and Fs homogenates could have affected fly performance on high salt food (see Discussion). Further experiments were performed to test the hypothesis that the increase in salt tolerance observed in Fs flies was at least partially due to changes in yeast microbiome, and to reveal specific characteristics of the microbiome that may improve salt tolerance in *Drosophila* lines reared on salty food.

### 3. Composition of the yeast microbiome

We inoculated the *Drosophila* homogenates from each of the four lines in ten Petri dishes (see Material and Methods) to assess quantitative and qualitative composition of the yeast microbiome. We used the same four homogenates as in the experiment described in the previous section. Five yeast species were detected in the homogenates:

1. *Pichia occidentalis* (in all four lines),
2. *Zygosaccharomyces bailii* (in Fn1 and Fn2),
3. *Starmerella bacillaris* (syn. *Candida zemplinina*; in Fs1 and Fs2),
4. *Candida californica* (Fs1),
5. *Pichia membranifaciens* (abundant in Fs2, a few cells in Fn1).

**Fig 4:**
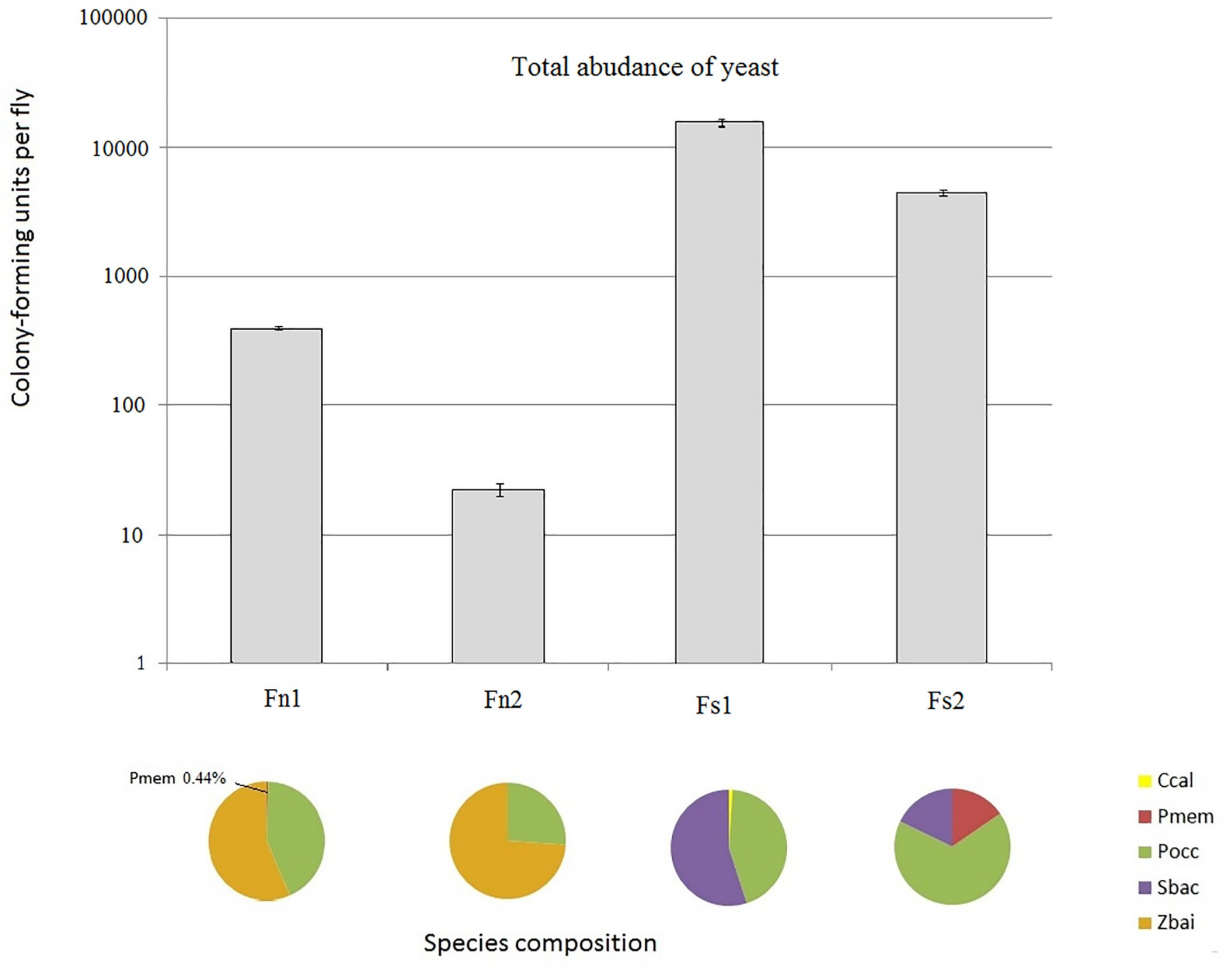
Mean number of colony-forming units (per fly, ± SEM) in homogenates of *D*. *melanogaster* from four experimental lines. Pie charts depict species composition of yeast in each line as percentages of the total abundance. Ccal, *Candida californica*; Pmem, *Pichia membranifaciens*; Pocc, *Pichia occidentalis*; Sbac, *Starmerella bacillaris*; Zbai, *Zygosaccharomyces bailii*.

The yeast microbiome composition is shown in Fig 4, raw data are available in S3 Table.

As depicted by the figure, the four fly lines differ greatly in the total abundance of yeast: from 22,3 CFU per fly in line Fn2 to 15393 CFU per fly in line Fs2. Altogether, the homogenates of the salt-tolerant flies (Fs1, Fs2) contain more yeasts than the homogenates of the control flies (Fn1, Fn2).

The species composition of yeasts also differ between the salt-tolerant and control flies. The most prominent differences concern *S*. *bacillaris* and *Z*. *bailii*. The former species is abundant in both salt-tolerant lines and absent in the control lines; the latter species, by contrast, is abundant in both control lines and absent in the salt-tolerant lines.

### 4. Influence of different yeast strains on reproductive efficiency of *D*. *melanogaster* on salty food

**Fig 5:**
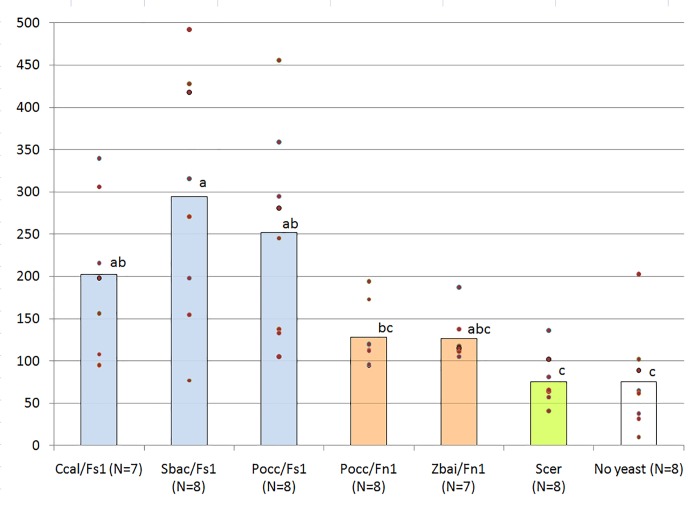
The influence of different yeast strains on reproductive efficiency of salt-naïve *D*. *melanogaster* on salty food. The vertical axis shows the mean number of adult offspring produced by 10 pairs of parents; individual data points are shown; bars depict mean values. Ccal, *Candida californica*; Pocc, *Pichia occidentalis*; Sbac, *Starmerella bacillaris*; Zbai, *Zygosaccharomyces bailii*; Scer, *Saccharomyces cerevisiae*; Fs1, yeast strains isolated from the salt-tolerant Fs1 flies; Fn1, yeast strains from the control line Fn1. N, number of vials in each test. Different letters indicate significant differences (p < 0.05, Dunn’s test).

The aim of the next experiment was to estimate the influence of the yeast strains present in the fly homogenates on the reproductive efficiency of the control (Fn1) flies on salty food. We chose three yeast strains extracted from the “salt-tolerant” Fs1 homogenate (*C*. *californica*, *S*. *bacillaris*, *P*. *occidentalis*) and two strains from the homogenate of the control line Fn1 (*P*. *occidentalis*, *Z*. *bailii*). Suspension of bakers’ yeast *Saccharomyces cerevisiae* and sterile water were used as controls. Thus, there were seven experimental conditions overall. The experiment was performed in November–December 2017. The results are shown in Fig 5, raw data are available in S4 Table.

The Kruskal–Wallis test allows us to reject the null hypothesis that all seven samples belong to the same distribution (*p* = 0.00005); *p*-values in the paired comparisons (Dunn’s test) are shown in Table 1.

**Table 1:** *P*-values in paired comparisons of seven experimental conditions (Dunn’s test). Abbreviations as in Fig 5.

	Ccal/Fs1	Sbac/Fs1	Pocc/Fs1	Pocc/Fn1	Zbai/Fn1	Scer
**Sbac/Fs1**	0.441998					
**Pocc/Fs1**	0.584940	0.817738				
**Pocc/Fn1**	0.259547	0.049664	0.083205			
**Zbai/Fn1**	0.303976	0.067178	0.107870	0.947533		
**Scer**	0.003542	0.000136	0.000338	0.064085	0.063652	
**No yeast**	0.003542	0.000136	0.000338	0.064085	0.063652	1.0

The results shown in Fig 5 and Table 1 agree with the assumption that yeast strains from the salt-tolerant Fs1 flies improve reproduction of salt-naïve flies on salty food compared to controls (bakers’ yeast and no yeast). At the same time, the positive effect of yeast strains from the control Fn1 flies is not significant. The strongest positive influence is shown by *S*. *bacillaris*, although differences between this strain and other yeast strains from the salt-tolerant flies are not statistically significant.

## Discussion

### 1. Acquired tolerance of *D*. *melanogaster* to high salt food medium

The food substrate with high (2% and more) NaCl concentration is an unfavourable (stressful) medium for *D*. *melanogaster*, because it induces high larval mortality and suppresses larval development. Typically, naïve adult *D*. *melanogaster* can endure up to 4% NaCl in the food medium. Above this concentration, flies continue to eat the food, but their lifespan shortens and fecundity decreases dramatically [20, 21]. Nevertheless, laboratory populations are able to adapt to NaCl concentrations up to 6–7% [20, 22] or even 7–8% [23, 24] over several dozen generations if the salt concentration increased gradually. Although there is some data on the mechanisms of short-term salt stress response in *Drosophila* [21], the nature of the flies’ long-term acquired tolerance to salt is not completely understood,.

The *D*. *melanogaster* lines Fs1 and Fs2, described in this study, have been reared on food with 4% NaCl since October 2014. Their reproductive efficiency on salty and normal food was tested for the first time 11 months after the start of the evolutionary experiment, in September 2015. We found that they produced more offspring on both types of food than the control flies reared on food N [26]. In the current study, we replicated this result three years after the start of the evolutionary experiment (Fig 2). It is difficult to compare directly the results of the first and second tests because the former was performed in test tubes with 10 mL of food and two pairs of parents, while the latter was performed in glass vials with 66.7 mL of food and ten pairs of parents. In any case, we have confirmed that the flies from lines Fs1 and Fs2 are more tolerant to high salt diet than the flies from lines Fn1 and Fn2 three years after the start of the evolutionary experiment (Fig 2), which makes it reasonable to search for the mechanisms of this adaptation.

### 2. Microbiota contributes to the observed increase in salt tolerance

We estimated the reproductive efficiency of the flies on salty food pre-inoculated with the homogenized salt-tolerant and control flies to assess the possible contribution of the microbiome to the adaptation of *D*. *melanogaster* to salty food. We have shown previously that pre-inoculation of the salty substrate with salt-tolerant homogenized flies reliably improved the fitness of *D*. *melanogaster* on food S. This result was obtained from the four *D*. *melanogaster* lines reared under different conditions (in population cages, with overlapping generations) [43, 44]. This apparently indicates that some quantitative or qualitative characteristics of the microbiome of the salt-tolerant flies, along with other features of the salt-tolerant fly homogenate, improve the reproductive efficiency of the flies on salty food. One of the goals of the current study was to verify the reproducibility of this result in the other four lines maintained under different conditions (in vials, with 20 randomly chosen flies transferred into a new vial every 14 days).

As shown in Fig 3, in the current study the previous result was replicated in general. Although not all the paired comparisons between the control and salt-tolerant lines showed statistically significant levels of difference, the directions of differences in all cases agreed with the expected ones. Therefore, the results are consistent with our hypothesis that microbiome contributes to the adaptation of *Drosophila* to salt. This makes it reasonable to search for specific characteristics of microbiome responsible for this contribution.

We acknowledge that additional factors independent of the different microbiome between Fn and Fs homogenate could have affected fly performance on high salt food pre-inoculated with different homogenates. Offspring number notably increased when the flies were given the Fn fly homogenate as compared to no homogenate (compare Fig 2 and Fig 3). This implies that flies likely fed on the homogenate for a while before fully relied on the fly food, and the nutritional content probably differed between the Fs and Fn homogenate. Moreover, the flies themselves could be producing factors that alleviate salt stress independently of their microbiome. This can confound the interpretation of the results.

In order to reduce this uncertainty, several experimental approaches can be used. One possible approach is to compare the effects of Fn and Fs homogenates to those of axenic flies homogenates, or to add antibiotics and fungicides to Fn and Fs homogenates in order to remove the microbiome. However, such procedures would inevitably lead to new complications, e.g., rearing flies in axenic conditions would certainly change their physiology and nutritional value, while adding fungicides to homogenates would disrupt the normal growth of yeasts on the surface of fly food after inoculation. To this end, we have used two other approaches. First, we compared yeast microbiome composition in fly homogenates and found substantial differences between Fn and Fs homogenates. Second, we evaluated the effects of pure cultures of different yeast strains isolated from Fn and Fs flies on the reproduction efficiency of naïve flies on high salt food, and found that strains isolated from Fs flies tend to have the strongest positive effect (although their advantage over the strains from Fn flies is not statistically significant). The results are compatible with the hypothesis that yeasts do contribute to the observed increase in salt tolerance in Fs flies, as discussed in the next two sections. Importantly, these results do not provide a basis for a claim that microbiome is the only source of the increased salt tolerance in the Fs flies. Other factors, including genetic and epigenetic changes in the flies themselves, may also contribute to the observed adaptation; further research is needed to elucidate the relative importance of different factors.

### 3. Yeast microbiome composition differs between the salt-tolerant and control *Drosophila* lines

The research of the host-microbe interactions on the *D*. *melanogaster* model is currently focused mostly on the gut bacteria [29–31, 33–38, 46, 47]. However, yeasts apparently form a very important part of the microbiome of *Drosophila* [10, 39–42, 48–50]. Yeasts affect different aspects of physiology, immune response, and behavior of *Drosophila*. For instance, some yeast species influence the larval survival and growth rate, along with the adult body mass; in addition, larvae are selective to yeasts and prefer the species that enhance larval growth [40–42]. *Drosophila* larvae and adults, on the other hand, can influence the species richness of yeast communities that develop on various food substrates [48]. At least some yeast species survive the passage through larval and adult *D*. *melanogaster* guts, making it possible for the flies to be the effective vectors of yeasts in the wild [10, 48–50].

In the current study, we focused on the possible impact of the yeast component of the microbiome of *D*. *melanogaster* on the flies' adaptation to salty food. We compared the yeast composition in four *Drosophila* lines and found that the control and salt-tolerant lines differ in the total amount of yeasts (salt-tolerant flies carry much more yeast cells) and species composition (Fig 4). Yeast *S*. *bacillaris* is present in the homogenates of the Fs1 and Fs2 flies, but absent in the homogenates of the Fn1 and Fn2 flies; *Z*. *bailii*, by contrast, is abundant in both control lines, but absent in the salt-tolerant lines.

It is interesting to compare these results with the ones from our previous study, where we performed a similar analysis in four other fly lines (two salt-adapted and two control ones) maintained under different conditions (in population cages). These lines have lower species diversity of yeasts than the ones kept in vials. We found only two abundant yeast species, *P*. *membranifaciens* and *S*. *bacillaris*, and only the distribution of the latter species was correlated with salt tolerance. *S*. *bacillaris* was abundant in both salt-tolerant lines and absent or scarce in both control lines [44].

Thus, in all eight experimental *D*. *melanogaster* lines tested to date (four analyzed in this study and four analyzed earlier [43, 44]) we see a common pattern: *S*. *bacillaris* is always abundant in the homogenates of salt-tolerant flies but absent or scarce in the homogenates of the control flies. At the same time, other parameters of the yeast microbiome (the total amount of yeast cells and relative abundance of species other than *S*. *bacillaris*) vary a lot and do not show apparent relationship with either salt-tolerant or control fly lines. It makes *S*. *bacillaris* a likely candidate for the role of the microbiome component that positively contributes to the *D*. *melanogaster* adaptation to salty food.

*S*. *bacillaris* is a genetically heterogeneous, psychrotolerant, osmotolerant, asporogenic species often found on grapes, in grape must and wine [51, 52]. It is sometimes recorded in soil, rotting watermelons and bananas; it was also found on *Drosophila* in the USA and Hungary, indicating that *Drosophila* can participate in the distribution of this yeast species [48, 52, 53]. It has been shown that *S*. *bacillaris* yeast is edible for *D*. *melanogaster* larvae, and that larvae fed on bananas encourage the consistent development of this yeast species along with two others (*Candida californica* and *Pichia kluvyeri*), while simultaneously discouraging the growth of filamentous fungi. Moreover, some *S*. *bacillaris* cells (as well as some cells of *C*. *californica* and *P*. *kluvyeri*) survive passing through the larval gut [48, 50]. As far as we know, there are no data on specific relationship of *S*. *bacillaris* with salty substrates, or with the development of *Drosophila* on these substrates. It should be noted that the cells of *S*. *bacillaris* are generally smaller than those of the other yeast species found in our fly lines. This fact may be related to the positive effect of *S*. *bacillaris* on fly fecundity on high salt food (see next section); further research is needed to clarify this point.

### 4. Yeast strains from salt-tolerant flies enhance the reproduction of *D*. *melanogaster* on salty food

Fig 5 depicts the results of the experiment in which we assessed the influence of five yeast strains (three strains extracted from the salt-tolerant Fs1 line and two strains from the control Fn1 line) on the reproductive efficiency of *D*. *melanogaster* on salty food. As controls, we used the baker’s yeast *S*. *cerevisiae* and sterile water.

We found that yeast strains extracted from salt-tolerant flies significantly improved the reproductive efficiency of salt-naïve *D*. *melanogaster* on salty food compared to both controls (Fig 5). Yeast strains extracted from control (naïve) flies may also have some positive effect, but in our experiment it was not statistically significant. As we expected, the strongest positive effect was from the yeast S. *bacillaris*, although its difference from two other strains extracted from the salt-tolerant flies did not reach the level of statistical significance.

*Drosophila* larvae can selectively improve the reproduction of some yeast species, including *S*. *bacillaris*, on their food substrate, at the same time preventing the reproduction of other fungi, which results in higher yeast community similarity in the presence of the larvae [48]. Further research is needed to find out if the interactions between *D*. *melanogaster* and particular yeast species are robust and specific enough to consider them as an example of agricultural symbiosis, such as the symbioses described in ants, termites, and some other animals [54, 55].

Remarkably, the baker’s yeast *S*. *cerevisiae*, which we used as one of the controls, did not have any noticeable effect on the reproduction of *D*. *melanogaster* on salty food, unlike yeasts extracted from salt-tolerant flies. This seems to agree with the conclusion from [10] that baker’s yeast, which is very rarely associated with *Drosophila* in the wild [39, 56], is not the best model for studying the *Drosophila*-yeast interactions, despite the fact that this species is often used in such studies (e.g., [50, 57]). It should be noted, however, that salt stress is not a natural condition for *D*. *melanogaster* (although it is for some other dipterans [28]), and thus the failure of *S*. *cerevisiae* to alleviate this stress does not, in itself, provide enough evidence to consider *D*. *melanogaster*–*S*. *cerevisiae* interaction a poor experimental model.

In our experiment, we did not measure the number of yeast cells used to inoculate food vials, but rather used the same volume of biomass for each yeast strain. As cell size of different yeast species vary substantially (e.g., *S*. *bacillaris* cells are generally smaller than those of the other species tested), we acknowledge that the different effects of yeast strains on fly reproduction may be partially due to different cell numbers in yeast inoculates. However, it is not evident that controling for cell number would be more appropriate than controlling for biomass. Whatever benefits the flies receive from the yeasts (e.g., nutrition, metabolites, or substrate transformation), these benefits may be more related to the biomass of the yeasts than to their cell numbers. For instance, yeasts are known to be a major food source for *Drosophila* in both adult and larval stages [42], yeast species with different cell sizes are palatable to *Drosophila* [48], and the nutritional value of ingested yeasts apparently depends more on biomass than on cell count. From the other hand, it is quite probable that the benefit received by the flies from *S*. *bacillaris* on salty food is related to the small size of the cells of this species. Further research is needed to clarify this issue.

In general, our results agree with the hypothesis that some components of yeast microbiome contribute, along with other possible factors, to the adaptation of *D*. *melanogaster* to salty food observed in experimental evolution studies. It means that the possible role of microbiome should be taken into account when interpreting the results of such studies. The generally accepted (although usually implicit) presumption that the observed adaptation is exclusively due to genetic or epigenetic changes of the experimental population of the studied macroorganisms can be misleading. It is probably more reasonable to study the *Drosophila* adaptation to adverse conditions at the holobiont level, as proposed by the proponents of the “hologenome theory of evolution.”

## Supporting information

S1 Table Reproduction efficiency of four *D*. *melanogaster* lines on foods N and S. (DOCX)

S2 Table The influence of the control and salt-tolerant fly homogenates on the reproductive efficiency of control flies on food S. (DOCX)

S3 Table Mean number of colony-forming units in homogenates of *D*. *melanogaster* from four experimental lines. (DOCX)

S4 Table The influence of different yeast strains on reproductive efficiency of salt-naïve *D*. *melanogaster* on salty food. (DOCX)
