# Supplementary material for: Yeasts affect tolerance of Drosophila melanogaster to food substrate with high NaCl concentration
Source: PLoS One. 2019 Nov 6;14(11):e0224811. doi: 10.1371/journal.pone.0224811 (PMC6834263; doi:10.1371/journal.pone.0224811)
Supplement: S1 Table — (DOCX) [file pone.0224811.s001.docx]

| S1 Table. Data for Figure 2 (Reproduction efficiency of four *D. melanogaster* lines on foods N and S) | | | | | |
| --- | --- | --- | --- | --- | --- |
|  |  |  |  |  |  |
| Left graph, Food N | | | Right graph, Food S | | |
|  |  |  |  |  |  |
| Line of flies | Vial number | Number of offspring | Line of flies | Vial number | Number of offspring |
| Fn1 | 1 | 557 | Fn1 | 1 | 97 |
| Fn1 | 2 | 471 | Fn1 | 2 | 33 |
| Fn1 | 3 | 562 | Fn1 | 3 | 77 |
| Fn1 | 4 | 654 | Fn1 | 4 | 135 |
| Fn1 | 5 | 557 | Fn1 | 5 | 56 |
| Fn1 | 6 | 703 | Fn2 | 1 | 46 |
| Fn1 | 7 | 572 | Fn2 | 2 | 60 |
| Fn1 | 8 | 597 | Fn2 | 3 | 67 |
| Fn1 | 9 | 579 | Fn2 | 4 | 59 |
| Fn1 | 10 | 528 | Fn2 | 5 | 18 |
| Fn2 | 1 | 422 | Fs1 | 1 | 138 |
| Fn2 | 2 | 438 | Fs1 | 2 | 255 |
| Fn2 | 3 | 467 | Fs1 | 3 | 138 |
| Fn2 | 4 | 492 | Fs1 | 4 | 102 |
| Fn2 | 5 | 464 | Fs1 | 5 | 128 |
| Fn2 | 6 | 452 | Fs1 | 6 | 163 |
| Fn2 | 7 | 434 | Fs1 | 7 | 141 |
| Fn2 | 8 | 472 | Fs1 | 8 | 117 |
| Fn2 | 9 | 502 | Fs1 | 9 | 253 |
| Fn2 | 10 | 455 | Fs1 | 10 | 223 |
| Fs1 | 1 | 576 | Fs2 | 1 | 267 |
| Fs1 | 2 | 597 | Fs2 | 2 | 81 |
| Fs1 | 3 | 617 | Fs2 | 3 | 123 |
| Fs1 | 4 | 674 | Fs2 | 4 | 101 |
| Fs1 | 5 | 661 | Fs2 | 5 | 95 |
| Fs2 | 1 | 776 | Fs2 | 6 | 231 |
| Fs2 | 2 | 695 | Fs2 | 7 | 234 |
| Fs2 | 3 | 731 | Fs2 | 8 | 301 |
| Fs2 | 4 | 658 | Fs2 | 9 | 203 |
| Fs2 | 5 | 573 | Fs2 | 10 | 137 |
