# Supplementary material for: Yeasts affect tolerance of Drosophila melanogaster to food substrate with high NaCl concentration
Source: PLoS One. 2019 Nov 6;14(11):e0224811. doi: 10.1371/journal.pone.0224811 (PMC6834263; doi:10.1371/journal.pone.0224811)
Supplement: S2 Table — (DOCX) [file pone.0224811.s002.docx]

| S2 Table. Data for Figure 3 (The influence of the control (Fn1, Fn2) and salt-tolerant (Fs1, Fs2) fly homogenates on the reproductive efficiency of Fn1 flies on food S) | | |
| --- | --- | --- |
|  |  |  |
| Homogenate type | Vial number | Number of offspring |
| Fn1 | 1 | 138 |
| Fn1 | 2 | 174 |
| Fn1 | 3 | 224 |
| Fn1 | 4 | 88 |
| Fn1 | 5 | 129 |
| Fn2 | 1 | 234 |
| Fn2 | 2 | 119 |
| Fn2 | 3 | 188 |
| Fn2 | 4 | 325 |
| Fn2 | 5 | 195 |
| Fs1 | 1 | 258 |
| Fs1 | 2 | 257 |
| Fs1 | 3 | 362 |
| Fs1 | 4 | 199 |
| Fs1 | 5 | 402 |
| Fs2 | 1 | 325 |
| Fs2 | 2 | 195 |
| Fs2 | 3 | 333 |
| Fs2 | 4 | 274 |
| Fs2 | 5 | 301 |
