# Supplementary material for: Yeasts affect tolerance of Drosophila melanogaster to food substrate with high NaCl concentration
Source: PLoS One. 2019 Nov 6;14(11):e0224811. doi: 10.1371/journal.pone.0224811 (PMC6834263; doi:10.1371/journal.pone.0224811)
Supplement: S3 Table — (DOCX) [file pone.0224811.s003.docx]

| S3 Table. Data for Figure 4 (Mean number of colony-forming units in homogenates of *D. melanogaster* from four experimental lines)^[[1]](#endnote-1)^ | | | | | | | |
| --- | --- | --- | --- | --- | --- | --- | --- |
|  |  |  |  |  |  |  |  |
| Homogenate type | Petri dish number | Number of colony-forming units (CFU) per fly | | | | | |
|  |  | Total | Species composition of yeasts (CFU) | | | | |
|  |  |  | *Candida californica* | *Pichia membranifaciens* | *Pichia occidentalis* | *Starmerella bacillaris* | *Zygosaccharomyces bailii* |
| Fn1 | 1 | 394 | 0 | 0 | 136 | 0 | 258 |
| Fn1 | 2 | 406 | 0 | 4 | 162 | 0 | 240 |
| Fn1 | 3 | 508 | 0 | 2 | 182 | 0 | 324 |
| Fn1 | 4 | 384 | 0 | 6 | 178 | 0 | 200 |
| Fn1 | 5 | 358 | 0 | 0 | 146 | 0 | 212 |
| Fn1 | 6 | 412 | 0 | 2 | 172 | 0 | 238 |
| Fn1 | 7 | 440 | 0 | 0 | 192 | 0 | 248 |
| Fn1 | 8 | 288 | 0 | 0 | 184 | 0 | 104 |
| Fn1 | 9 | 386 | 0 | 0 | 154 | 0 | 232 |
| Fn1 | 10 | 418 | 0 | 4 | 160 | 0 | 254 |
| Fn2 | 1 | 14 | 0 | 0 | 4 | 0 | 10 |
| Fn2 | 2 | 18 | 0 | 0 | 8 | 0 | 10 |
| Fn2 | 3 | 24 | 0 | 0 | 4 | 0 | 20 |
| Fn2 | 4 | 20 | 0 | 0 | 4 | 0 | 16 |
| Fn2 | 5 | 16 | 0 | 0 | 4 | 0 | 10 |
| Fn2 | 6 | 16 | 0 | 0 | 2 | 0 | 14 |
| Fn2 | 7 | 24 | 0 | 0 | 8 | 0 | 16 |
| Fn2 | 8 | 26 | 0 | 0 | 8 | 0 | 18 |
| Fn2 | 9 | 22 | 0 | 0 | 8 | 0 | 14 |
| Fn2 | 10 | 42 | 0 | 0 | 10 | 0 | 32 |
| Fs1 | 1 | 12320 | 120 | 0 | 5200 | 7000 | 0 |
| Fs1 | 2 | 14510 | 170 | 0 | 8540 | 5800 | 0 |
| Fs1 | 3 | 13346 | 146 | 0 | 6800 | 6400 | 0 |
| Fs1 | 4 | 12920 | 120 | 0 | 5400 | 7400 | 0 |
| Fs1 | 5 | 14624 | 200 | 0 | 6424 | 8000 | 0 |
| Fs1 | 6 | 15570 | 170 | 0 | 6600 | 8800 | 0 |
| Fs1 | 7 | 18848 | 48 | 0 | 8200 | 10600 | 0 |
| Fs1 | 8 | 16020 | 220 | 0 | 7000 | 8800 | 0 |
| Fs1 | 9 | 15370 | 170 | 0 | 5600 | 9600 | 0 |
| Fs1 | 10 | 20400 | 200 | 0 | 7400 | 12800 | 0 |
| Fs2 | 1 | 3198 | 0 | 774 | 2224 | 200 | 0 |
| Fs2 | 2 | 4440 | 0 | 600 | 3240 | 600 | 0 |
| Fs2 | 3 | 3500 | 0 | 600 | 2240 | 660 | 0 |
| Fs2 | 4 | 3610 | 0 | 520 | 2250 | 840 | 0 |
| Fs2 | 5 | 4304 | 0 | 720 | 2024 | 1560 | 0 |
| Fs2 | 6 | 4820 | 0 | 600 | 3220 | 1000 | 0 |
| Fs2 | 7 | 4860 | 0 | 620 | 3240 | 1000 | 0 |
| Fs2 | 8 | 5424 | 0 | 800 | 4024 | 600 | 0 |
| Fs2 | 9 | 4624 | 0 | 800 | 3224 | 600 | 0 |
| Fs2 | 10 | 5440 | 0 | 600 | 4040 | 800 | 0 |

1. The amount of homogenate used per plate was equal to 0.5 homogenized flies. [↑](#endnote-ref-1)
