# Supplementary material for: Yeasts affect tolerance of Drosophila melanogaster to food substrate with high NaCl concentration
Source: PLoS One. 2019 Nov 6;14(11):e0224811. doi: 10.1371/journal.pone.0224811 (PMC6834263; doi:10.1371/journal.pone.0224811)
Supplement: S4 Table — (DOCX) [file pone.0224811.s004.docx]

| S4 Table. Data for Figure 5 (The influence of different yeast strains on reproductive efficiency of salt-naïve *D. melanogaster* on salty food) | | |
| --- | --- | --- |
|  |  |  |
| Experimental conditions (yeast species//extracted from fly line) | Vial number | Number of offspring |
| *Candida californica//*Fs1 | 1 | 306 |
| *Candida californica//*Fs1 | 2 | 156 |
| *Candida californica//*Fs1 | 3 | 198 |
| *Candida californica//*Fs1 | 4 | 340 |
| *Candida californica//*Fs1 | 5 | 95 |
| *Candida californica//*Fs1 | 6 | 216 |
| *Candida californica//*Fs1 | 7 | 108 |
| *Starmerella bacillaris//*Fs1 | 1 | 492 |
| *Starmerella bacillaris//*Fs1 | 2 | 428 |
| *Starmerella bacillaris//*Fs1 | 3 | 418 |
| *Starmerella bacillaris//*Fs1 | 4 | 316 |
| *Starmerella bacillaris//*Fs1 | 5 | 271 |
| *Starmerella bacillaris//*Fs1 | 6 | 198 |
| *Starmerella bacillaris//*Fs1 | 7 | 155 |
| *Starmerella bacillaris//*Fs1 | 8 | 77 |
| *Pichia occidentalis/*/Fs1 | 1 | 105 |
| *Pichia occidentalis/*/Fs1 | 2 | 456 |
| *Pichia occidentalis/*/Fs1 | 3 | 281 |
| *Pichia occidentalis/*/Fs1 | 4 | 359 |
| *Pichia occidentalis/*/Fs1 | 5 | 138 |
| *Pichia occidentalis/*/Fs1 | 6 | 295 |
| *Pichia occidentalis/*/Fs1 | 7 | 133 |
| *Pichia occidentalis/*/Fs1 | 8 | 245 |
| *Pichia occidentalis//*Fn1 | 1 | 120 |
| *Pichia occidentalis//*Fn1 | 2 | 194 |
| *Pichia occidentalis//*Fn1 | 3 | 95 |
| *Pichia occidentalis//*Fn1 | 4 | 120 |
| *Pichia occidentalis//*Fn1 | 5 | 113 |
| *Pichia occidentalis//*Fn1 | 6 | 96 |
| *Pichia occidentalis//*Fn1 | 7 | 112 |
| *Pichia occidentalis//*Fn1 | 8 | 173 |
| *Zygosaccharomyces bailii//*Fn1 | 1 | 112 |
| *Zygosaccharomyces bailii//*Fn1 | 2 | 118 |
| *Zygosaccharomyces bailii//*Fn1 | 3 | 115 |
| *Zygosaccharomyces bailii//*Fn1 | 4 | 187 |
| *Zygosaccharomyces bailii//*Fn1 | 5 | 111 |
| *Zygosaccharomyces bailii//*Fn1 | 6 | 105 |
| *Zygosaccharomyces bailii//*Fn1 | 7 | 138 |
| *Saccharomyces cerevisiae* | 1 | 64 |
| *Saccharomyces cerevisiae* | 2 | 41 |
| *Saccharomyces cerevisiae* | 3 | 102 |
| *Saccharomyces cerevisiae* | 4 | 136 |
| *Saccharomyces cerevisiae* | 5 | 66 |
| *Saccharomyces cerevisiae* | 6 | 81 |
| *Saccharomyces cerevisiae* | 7 | 57 |
| *Saccharomyces cerevisiae* | 8 | 57 |
| No yeast | 1 | 203 |
| No yeast | 2 | 102 |
| No yeast | 3 | 89 |
| No yeast | 4 | 65 |
| No yeast | 5 | 62 |
| No yeast | 6 | 38 |
| No yeast | 7 | 32 |
| No yeast | 8 | 10 |
